# Autoantibodies against complement factor B in rheumatoid arthritis

**DOI:** 10.3389/fimmu.2023.1113015

**Published:** 2023-02-20

**Authors:** Alexandra T. Matola, Angéla Fülöp, Bernadette Rojkovich, György Nagy, Gabriella Sármay, Mihály Józsi, Barbara Uzonyi

**Affiliations:** ^1^ Department of Immunology, ELTE Eötvös Loránd University, Budapest, Hungary; ^2^ MTA-ELTE Complement Research Group, Eötvös Loránd Research Network (ELKH) at the Department of Immunology, ELTE Eötvös Loránd University, Budapest, Hungary; ^3^ Buda Hospital of the Hospitaller Order of Saint John of God, Budapest, Hungary; ^4^ Department of Rheumatology and Clinical Immunology, Semmelweis University, Budapest, Hungary; ^5^ Department of Internal Medicine and Oncology, Semmelweis University, Budapest, Hungary; ^6^ Heart and Vascular Center, Faculty of Medicine, Semmelweis University, Budapest, Hungary; ^7^ Department of Genetics, Cell- and Immunobiology, Faculty of Medicine, Semmelweis University, Budapest, Hungary

**Keywords:** complement, alternative pathway, C3 convertase, autoantibody, rheumatoid arthritis, factor B (FB)

## Abstract

Rheumatoid arthritis (RA) is a chronic inflammatory autoimmune disorder affecting the joints. Many patients carry anti-citrullinated protein autoantibodies (ACPA). Overactivation of the complement system seems to be part of the pathogenesis of RA, and autoantibodies against the pathway initiators C1q and MBL, and the regulator of the complement alternative pathway, factor H (FH), were previously reported. Our aim was to analyze the presence and role of autoantibodies against complement proteins in a Hungarian RA cohort. To this end, serum samples of 97 ACPA-positive RA patients and 117 healthy controls were analyzed for autoantibodies against FH, factor B (FB), C3b, C3-convertase (C3bBbP), C1q, MBL and factor I. In this cohort, we did not detect any patient with FH autoantibodies but detected C1q autoantibodies in four patients, MBL autoantibodies in two patients and FB autoantibodies in five patients. Since the latter autoantibodies were previously reported in patients with kidney diseases but not in RA, we set out to further characterize such FB autoantibodies. The isotypes of the analyzed autoantibodies were IgG2, IgG3, IgGκ, IgGλ and their binding site was localized in the Bb part of FB. We detected *in vivo* formed FB–autoanti-FB complexes by Western blot. The effect of the autoantibodies on the formation, activity and FH-mediated decay of the C3 convertase in solid phase convertase assays was determined. In order to investigate the effect of the autoantibodies on complement functions, hemolysis assays and fluid phase complement activation assays were performed. The autoantibodies partially inhibited the complement-mediated hemolysis of rabbit red blood cells, inhibited the activity of the solid phase C3-convertase and C3 and C5b-9 deposition on complement activating surfaces. In summary, in ACPA-positive RA patients we identified FB autoantibodies. The characterized FB autoantibodies did not enhance complement activation, rather, they had inhibitory effect on complement. These results support the involvement of the complement system in the pathomechanism of RA and raise the possibility that protective autoantibodies may be generated in some patients against the alternative pathway C3 convertase. However, further analyses are needed to assess the exact role of such autoantibodies.

## Introduction

Rheumatoid arthritis (RA) is a chronic inflammatory autoimmune disorder targeting typically the peripheral joints of the hands and feet, leading to persistent inflammation. With time it may cause the formation of a new tissue with infiltrating inflammatory immune cells called pannus and the destruction of the bones and cartilage which may give rise to deformities (1, 2). RA affects approximately 0.5-1% of the population (3) with slightly different frequencies between geographical regions and populations (4–6). RA affects mainly women with rising prevalence with age (7, 8). Though the cause of RA is unknown, several predisposing factors may play a role in disease development: environmental exposures such as smoking (9), infection and genetic factors (3, 10, 11). Presumably, hormonal factors also play a role in the mechanism causing the overrepresentation of women in the disease (8, 12).

Most of the patients develop rheumatoid factor or anti-modified protein antibodies. The classical serological marker of RA was rheumatoid factor, an autoantibody targeting the Fc region of IgG. It has relatively low specificity as it can be found in other pathological conditions and healthy individuals (8, 13, 14). By contrast, autoantibodies against modified (e.g., carbamylated, citrullinated, acetylated) self-proteins are more specific for RA and can be detected in patients before clinical onset, in the subclinical stage (8, 15). The most frequently detected anti-citrullinated protein antibody (ACPA) is a diagnostic criterion of RA (16) and the presence of ACPA can predict the development of the disease (17–19).

The above-mentioned autoantibodies can form immune complexes with self-antigens in the patients and activate complement, which plays a remarkable role in the pathogenesis of RA (20). The complement system as part of the innate immune system has a crucial role in host defense including elimination of microbes, apoptotic and necrotic cells, and the clearance of immune complexes (21). The activation trigger defines by which of the three pathways will complement be activated. The classical pathway is initiated by the binding of its pattern recognition molecule C1q to IgG- or IgM-containing immune complexes. The lectin pathway is induced by sensing microbial carbohydrate structures, and the alternative pathway is constantly activated on a low level (C3 tick over mechanism) due to the spontaneous hydrolysis of the thioester group of the central C3 molecule (C3(H_2_O)). Thus, factor B (FB) bound to C3(H_2_O) and cleaved by factor D (FD), forms the fluid phase C3 convertase of the alternative pathway, C3(H_2_O)Bb. This in turn cleaves C3, generating C3b which can covalently bind to target surfaces and together with FB (again, cleaved by FD) and the stabilizing properdin forms the C3 convertase of the alternative pathway C3bBbP. The activation of the cascades leads to the generation of small anaphylatoxins (e.g., C3a, C5a) and larger fragments which opsonize the target (20–23). To avoid activation on host surfaces, complement is tightly regulated at several points by membrane-bound and fluid-phase regulators (24). In case of mutations or presence of autoantibodies against complement components and/or regulators, the system turns defective, and plays an important role in the pathomechanism of several autoimmune diseases (20, 25).

The involvement of the complement system in RA has long been studied. Decreased levels of C3 and C4 proteins (26) and elevated levels of complement activation products C3a, C5a, Bb and soluble C5b-9 (sC5b-9) in the synovial fluid of patients suggested the overactivation of the complement system (27–29). Elevated levels of sC5b-9 in patients’ plasma (30, 31) indicate that the complement activation in RA is not limited to the joints but may affect other organs and tissues. Furthermore, treatment with anti-rheumatic drugs can decrease the elevated sC5b-9 levels (31). Autoantibodies against complement components were found in RA patients, including C1q (32, 33), factor H (FH) (34) and mannose binding lectin (MBL) (35), further confirming the involvement of the three complement pathways in the pathomechanism of RA, although the functions and relevance of these autoantibodies were not investigated and are still unknown.

To assess the role of anti-complement autoantibodies in RA, the aim of this study was to investigate whether the previously reported autoantibodies can be detected and/or other, novel autoantibodies can be found in a Hungarian cohort of RA patients. Here, we report the identification and characterization of FB autoantibodies in RA.

## Materials and methods

### Proteins and antibodies

Complement proteins FH (#341274), FB (#341262), C3b (#204860), C3 (#204885), factor I (FI) (#341280), C1q (#204876), goat anti-FB (#341272) and goat anti-FH (#341276) antiserum were obtained from Merck (Darmstadt, Germany; obtained *via* Merck Life Science Kft., Budapest, Hungary). FD (#A409), properdin (#A412), mouse anti-C5b-9 (#A239), anti-FB (Bb) (#A227), anti-FB (Ba) (#A225) and C3a EIA kit (#A032) were from Quidel (obtained *via* Biomedica, Budapest, Hungary). Purified human IgG (#I2511), human serum albumin (#A3782), human alpha-1 antitrypsin (#SRP6312), antibodies against C1q (#234390), human IgG (#A6029), IgM (#A0420), IgA (#A0295), IgG1 (clone HP-6001, #I9388), IgG2 (clone HP-6002, #I9513), IgG3 (clone HP-6050, #I7260), IgG4 (clone HP-6025, #I7385), IgGκ (clone KP-53, #K4377), IgGλ (clone HP-6054, #L6522), and avidin-HRP (#A7419) were purchased from Merck. Mouse IgG1 (clone MOPC-21, #BZ-400101) was obtained from BioLegend (San Diego, CA). MBL (#9086-MB) and biotin-conjugated goat anti-MBL (#BAF2307) were from R&D Systems (Minneapolis, MN), HRP-conjugated goat anti-mouse (#P0447), rabbit anti-goat (#P0449) and swine anti-rabbit (#P0217) antibodies were from DAKO (Hamburg, Germany). Goat anti-C3 F(ab’)_2_ (#55062) and HRP-conjugated goat anti-C3 F(ab’)_2_ (#55237) were from MP Biomedicals (Solon, OH).

### Serum samples

Serum samples were collected from Hungarian rheumatoid arthritis patients (n=97) and from healthy individuals (n=117) after informed consent in accordance with the Declaration of Helsinki. The study was approved by the National Ethical Committee (33986-1/2018 EKU). Characteristics of the patients and healthy donors are shown in **Table 1**.

**Table 1:** Characteristics of patients with RA and healthy controls.

Characteristics		RA (N=97)	Healthy control (N=117)
Sex	Female (%)	79 (81.4%)	73 (62.4%)
	Male (%)	18 (18.6%)	44 (37.6%)
Age	Average (years)	62.5	31.2
	Median (years)	64	25
	Min-Max (years)	24-87	21-81

### Autoantibody detection

Autoantibodies were detected using ELISA method, as described by Józsi and Uzonyi (36). Briefly, FH, FB, C3b, FI in 5 µg/ml, MBL and C1q in 2 µg/ml and HSA or alpha-1 antitrypsin as negative controls were immobilized on microtiter plates. After blocking with 5% BSA in DPBS-0.1% Tween-20, serum samples were added in DPBS-0.1% Tween-20 at a dilution of 1:50 for 1 hour at RT. To detect autoantibodies against solid phase C3 convertase of the alternative pathway (C3bBbP), the convertase was built up (see below) and incubated with serum samples diluted 1:50. The bound autoantibodies were detected with HRP-conjugated goat anti-human IgG, anti-human IgA or anti-human IgM. Color reaction was developed by adding TMB solution (BioLegend). After stopping with H_2_SO_4_, absorbance was measured at 450 nm and at 620 nm as reference wavelength. To detect anti-MBL autoantibodies, serum samples were diluted in DPBS containing 10 mM EDTA. In the case of detecting anti-C1q autoantibodies, serum samples were diluted in DPBS containing 1 M NaCl to avoid interaction of C1q globular heads with IgG Fc parts. Samples were accepted as positive if the mean OD value for the investigated protein was at least two-fold greater than the mean OD value for the negative control protein alpha-1 antitrypsin. The isotypes of the autoantibodies were determined using monoclonal antibodies specific for IgG1, IgG2, IgG3, IgG4, IgGκ and IgGλ.

### IgG isolation

IgG fraction of the serum samples were isolated by Protein G column (GE Healthcare) according to the manufacturer’s instructions. For functional analyses, the *in vivo* formed FB–FB-autoantibody complexes were removed. To this end, FB-specific antibodies isolated by Protein G column from goat anti-FB antiserum were immobilized on NHS column (GE Healthcare). The patient’s and control’s IgG fractions were loaded onto the anti-FB column, the flow-through fraction was collected and tested for the presence of FB.

### Western blot

To analyze FHR1- and/or FHR3-deficiency, serum samples at a dilution of 1:50 were separated on 10% SDS-PAGE under non-reducing conditions and transferred to nitrocellulose membrane (BioRad). After blocking with 1% BSA, 4% non-fat milk powder in DPBS-0.05% Tween-20, FHR3 and FHR1 were detected with rabbit anti-FHR3 (kindly provided by Dr. Ilse Jongerius, Sanquin Health Solutions, Amsterdam, the Netherlands) and goat anti-FH antisera and the corresponding HRP-conjugated secondary antibodies, respectively. The blots were developed with the ECL detection kit (Merck).

To detect *in vivo* formed immune complexes, IgG fractions of the autoanti-FB positive patient and healthy controls were separated on 10% SDS-PAGE under non-reducing conditions and blotted onto nitrocellulose membrane. After blocking with 1% BSA, 4% non-fat milk powder in DPBS-0.05% Tween-20, the membranes were incubated with goat anti-FB antiserum and HRP-conjugated rabbit anti-goat antibodies. These immune complexes were removed before further experiments to prevent their interference with the assays.

### Convertase assays

The solid phase AP C3 convertase was built up as described by Hourcade et al. in 2002 (37) with modifications. C3b was immobilized at 5 µg/ml on microplate wells (Maxisorp, NUNC). After washing, 2 µg/ml FB, 4 µg/ml properdin and 0.1 µg/ml FD were added in convertase buffer (DPBS containing 4% BSA, 2 mM NiCl_2_, 0.1% Tween-20) for 1 hour at 37°C. Convertase formation was detected with goat anti-FB and HRP-conjugated rabbit anti-goat antibodies. Convertase activity was determined by adding 10 µg/ml C3 for 1 hour at 37°C. The generated C3a was measured by the C3a EIA kit (Quidel). In some assays, patient’s or control IgG was added together with convertase components to analyze their effect on convertase formation. Convertase decay was determined after incubation of the formed convertase with the isolated IgG fractions alone or in the presence of 1 µg/ml FH for 1 hour at 37°C. Remaining convertase was detected with goat anti-FB antiserum.

### Complement activation assay

LPS (#L4524, Merck) was immobilized at 10 µg/ml on Maxisorp microplate wells. Wells were washed with DPBS and blocked with 2% BSA. 10% complement active normal human serum (Quidel) was preincubated with the FB-free autoantibodies or with 5×10^6^ rabbit red blood cells (RRBC, Culex Bt.) as positive control for 15 min at 37°C. The serum was then added to the LPS-coated wells for 1 h at 37°C to determine the remaining complement activity. Complement deposition was measured with HRP-conjugated goat anti-C3 or mouse anti-C5b-9 and HRP-conjugated goat anti-mouse IgG.

### Hemolysis assay

5×10^6^ RRBC were incubated with 7% complement active normal human serum (Quidel) in HEPES buffer (20 mM HEPES, 7 mM MgCl_2_, 10 mM EGTA, 144 mM NaCl, 1% BSA, pH 7.4) with serial dilutions of purified IgG for 30 min at 37°C. After centrifugation, optical density of the supernatants was measured at 414 nm.

### Statistical analysis

Statistical analyses were performed with GraphPad Prism version 6.01 for Windows (GraphPad Software, San Diego, CA). A *p* value < 0.05 was considered statistically significant. Comparison of gender distribution between RA patients and healthy controls were determined by χ^2^ test. To compare continuous variables (age), Mann–Whitney U test was performed. Comparison of FHR1- and/or FHR3-deficiency distribution between RA patients and healthy donors were performed by χ^2^ test. For comparison of the effects of IgG isolated from RA547 or healthy controls, one-way ANOVA was used with Dunnett’s multiple comparison test.

## Results

### Screening for anti-complement autoantibodies

To investigate the presence and role of complement-specific autoantibodies in RA, we screened a Hungarian RA cohort for autoantibodies against FH, as well as C1q and MBL which were described as autoantibody targets in RA (33–35). In addition, we looked for autoantibodies against FB, FI, C3b and the solid phase C3 convertase C3bBbP, and analyzed the cohort for deficiency of the complement proteins FHR1 and FHR3. Serum samples of RA patients and healthy controls were tested for IgG and IgM antibodies against the above-mentioned complement components in ELISA.

**Figure 1:**
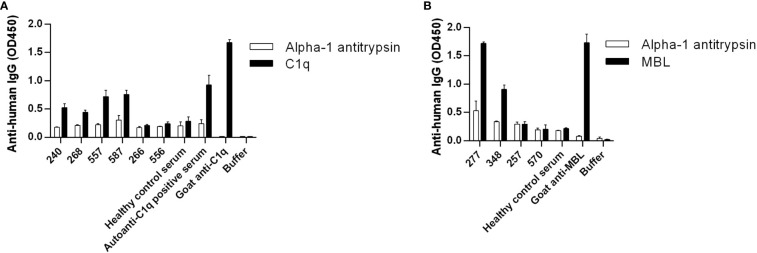
Detection of autoantibodies against C1q and MBL in RA patients. **(A)** C1q, **(B)** MBL, and alpha-1 antitrypsin as negative control were immobilized on microtiter plate wells. After blocking, serum samples diluted 1:50 were added. In the case of C1q, high salt concentration buffer was used and in the case of MBL 10 mM EDTA was used to exclude unspecific binding, as described in Materials and methods. Bound IgG was detected by HRP-conjugated anti-human IgG. Selected samples are shown: positive patient samples, some negative patient samples and controls. For full datasets please refer to **Supplementary Figure 1**. Data are mean ± SEM of two experiments.

**Figure 2:**
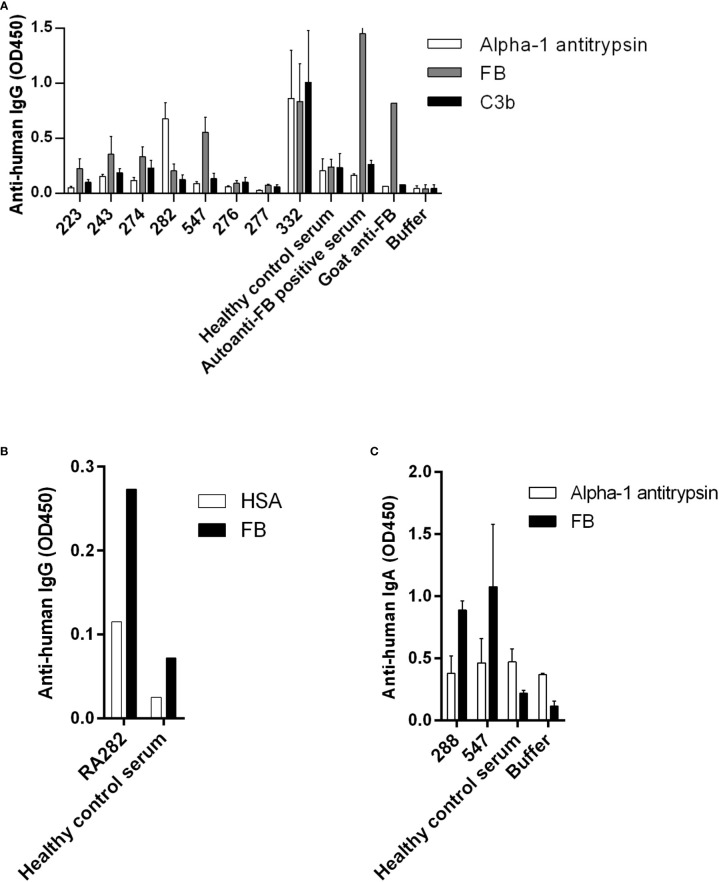
Autoanti-FB in RA patients. **(A)** FB, C3b and alpha-1 antitrypsin as negative control were immobilized on microtiter plate wells. After blocking, serum samples diluted 1:50 were added. Bound IgG was detected by HRP-conjugated anti-human IgG. Data are mean ± SEM of three experiments. **(B)** RA282 autoanti-FB was analyzed and confirmed using another negative control protein, human serum albumin (HSA). Data from a single experiment are shown. **(C)** IgA autoantibodies against FB were detected using HRP-conjugated anti-human IgA. Data are mean ± SD of two experiments. In all three panels selected samples are shown: positive patient samples, some negative patient samples and controls. For full datasets please refer to **Supplementary Figure 1**.

Healthy control samples were negative for all tested antibodies, and we did not detect autoantibodies against FH, FI, C3b or the C3bBbP convertase in this RA cohort (**Supplementary Figures 1C, D** and data not shown). Four patients (RA240, RA268, RA557, RA587) were positive for autoantibodies against C1q (**Figure 1A**; **Supplementary Figure 1A**), two patients were positive for autoantibodies against MBL (RA277, RA348) (**Figure 1B**; **Supplementary Figure 1B**) and five patients (RA223, RA243, RA274, RA282, RA547) were positive for autoanti-FB IgG (**Figure 2A**; **Supplementary Figure 1C**). The serum of patient RA282 showed high signal on alpha-1 antitrypsin, and it turned out that this patient had autoantibodies against alpha-1 antitrypsin. Positivity for FB-autoantibodies was determined using other inert proteins as negative controls (**Figure 2B**). In two patients (RA288, RA547) autoanti-FB IgA was detected (**Figure 2C**). Characteristics of the autoantibody positive patients are shown in **Table 2**. The titer and isotype were determined for all the five autoanti-FB IgG positive samples. We could detect in all samples both light chains and various combinations of heavy chains, pointing to an oligoclonal origin of the autoantibodies (**Table 3**). Based on the amount of serum available, RA547 sample was selected and further characterized in detail.

**Table 2:** Characteristics of the autoantibody positive patients.

Patient ID	Age at onset (years)	Sex	CRP (mg/l)	RF (U/ml)	Anti-CCP(U/ml)	DAS index	Treatment	Detected autoantibody
**223**	57	male	16	71	749	6.9	Methotrexat, Metilprednizolon, Sulfasalazine	anti-FB IgG
**240**	52	female			128.47	2.27		anti-C1q IgG
**243**	75	male		360.9	2403.87	0.84	Tocilizumab	anti-FB IgG
**268**	67	female				6.59		anti-C1q IgG
**274**	32	female		40	373	6.54	Methotrexat	anti-FB IgG
**277**	52	female		120	452.76	5.25	Leflunomide	anti-MBL IgG
**282**	80	male			497.89	3.09	Certolizumab pegol	anti-FB IgG
**288**	73	female		249	399	0.91	Tocilizumab	anti-FB IgA
**348**	73	female			617.34			anti-MBL IgG
**547**	41	female				3.0	Certolizumab pegol	anti-FB IgG, IgA
**557**	64	female			290	5.4		anti-C1q IgG
**587**	45	male			61			anti-C1q IgG

Reference ranges:

RF (rheumatoid factor): >20 U/ml positive.

Anti-CCP (cyclic citrullinated peptide): <20 non-RA, >20 RA.

DAS index (disease activity score): <2.6 remission, 2.6-3.1 low disease activity, 3.1-5.1 moderate disease activity, >5.1 high disease activity.

**Table 3:** Titer and isotype of the autoanti-FB IgG antibodies.

Sample ID	Titer	Isotype
RA223	1/800	IgG3, IgG4, IgGκ, IgGλ
RA243	1/200	IgG4, IgGκ, IgGλ
RA274	1/400	IgG2, IgG3, IgG4, IgGκ, IgGλ
RA282	1/50	IgG2, IgG4, IgGκ, IgGλ
RA547	1/800	IgG2, IgG3, IgGκ, IgGλ

**Figure 3:**
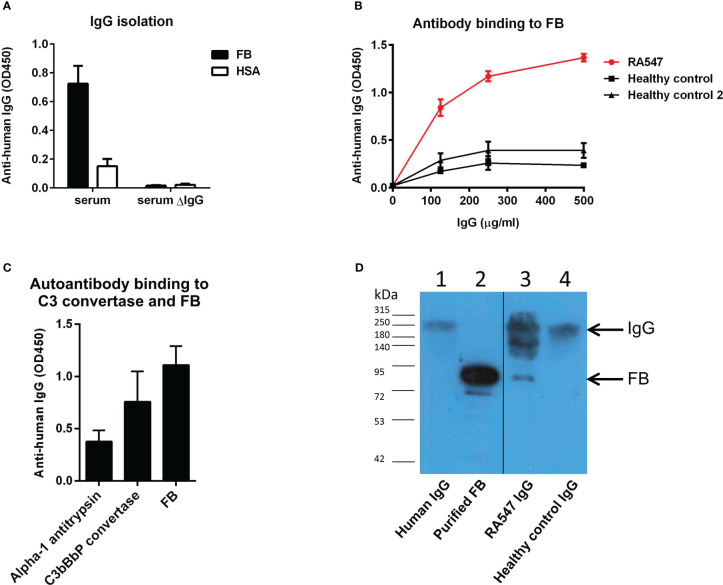
Anti-FB autoantibody characterization. **(A)** IgG was isolated with Protein G column from RA547 patient’s serum and healthy controls’ sera. The IgG depleted serum lost its ability to bind FB. **(B)** Serial dilutions from RA patient’s IgG showed dose-dependent binding to FB in contrast to healthy control IgG. Data are mean ± SD from three independent experiments. **(C)** Autoanti-FB bound to FB as part of the C3 convertase of the alternative pathway (C3bBbP). **(D)** *In vivo* formed IgG-FB complexes were detected in the patient’s IgG fraction with polyclonal anti-FB antibody in Western blot. Lanes: 1: purified IgG, 2: purified FB, 3: RA547 IgG, 4: Healthy control IgG. 25 µg isolated IgG was run on SDS-PAGE, blotted onto nitrocellulose membrane, and developed by anti-FB antibody. Blot is representative of three experiments.

IgG of RA547 and that of healthy controls was isolated from serum by Protein G column. IgG-depleted serum of RA547 did not show binding to FB (**Figure 3A**) while the isolated IgG fraction showed dose-dependent binding to FB (**Figure 3B**), and the antibody also bound to FB as part of the solid phase C3 convertase (C3bBbP) (**Figure 3C**). To test whether the antibody binds FB in its native form *in vivo*, the isolated IgG fraction was analyzed by Western blot. A strong band corresponding to FB was detected in the RA547 sample, indicating the formation of FB–anti-FB immune complexes *in vivo*. (**Figure 3D**).

### RA547 autoanti-FB characterization

**Figure 4:**
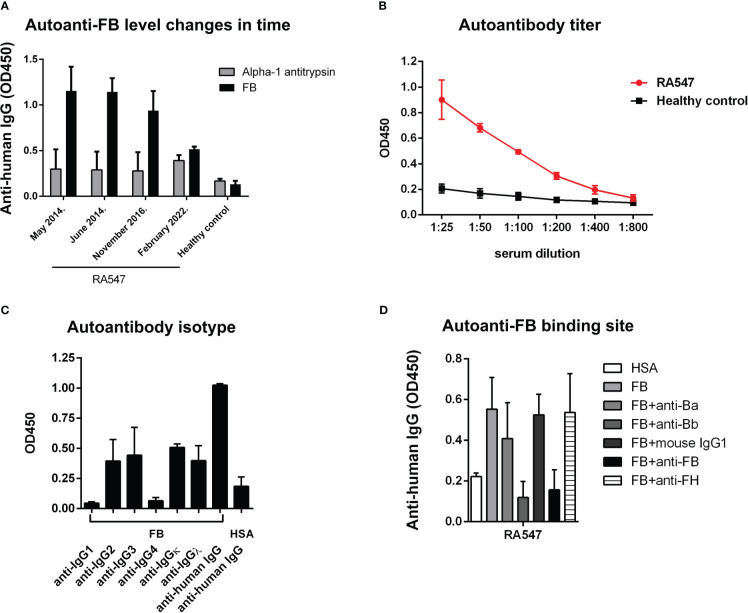
Autoantibody characterization. **(A)** We had serum samples from four time points to analyze the presence of autoanti-FB in RA547. Data are results ± SD of two experiments. **(B)** Titer of autoanti-FB was determined in ELISA, with serial dilutions of the patient’s serum. **(C)** Autoantibodies were added to immobilized FB, and the isotypes were determined with monoclonal antibodies against the IgG subclasses. Data are mean ± SD from two independent experiments. Values obtained with an autoantibody negative healthy control as a background were subtracted from the values obtained with RA547 before plotting. **(D)** Autoantibody binding to immobilized FB was decreased by the preincubation of FB with monoclonal anti-Bb antibody and with polyclonal anti-FB antiserum. Data are mean ± SEM from three independent experiments. Values obtained with an autoantibody negative healthy control as a background were subtracted from the values obtained with RA547.

Characteristics as titer, isotype, and binding site of the autoanti-FB in RA547 were determined in ELISA-based assays (**Table 3** and **Figure 4**). The titer and isotype were determined in the third available sample (**Figures 4B, C**). The presence of anti-FB autoantibodies was monitored in serial samples; over time, probably due to therapy, the anti-FB autoantibody levels decreased, and in the last sample it was not detectable anymore (**Figure 4A**). To determine the binding site of autoantibodies on FB, immobilized FB was preincubated with monoclonal antibodies specific for either the Ba or Bb part of FB, as well as with anti-FB antiserum. The anti-Bb and the polyclonal anti-FB reduced the binding of the autoantibodies to FB, while anti-Ba did not significantly influence the binding, suggesting that the autoantibody binding site is on the enzymatically active Bb part of FB (**Figure 4D**). Functional effects of the antibodies were analyzed in further assays.

### FB autoantibodies influence the activation of the alternative pathway and affect the terminal pathway

**Figure 5:**
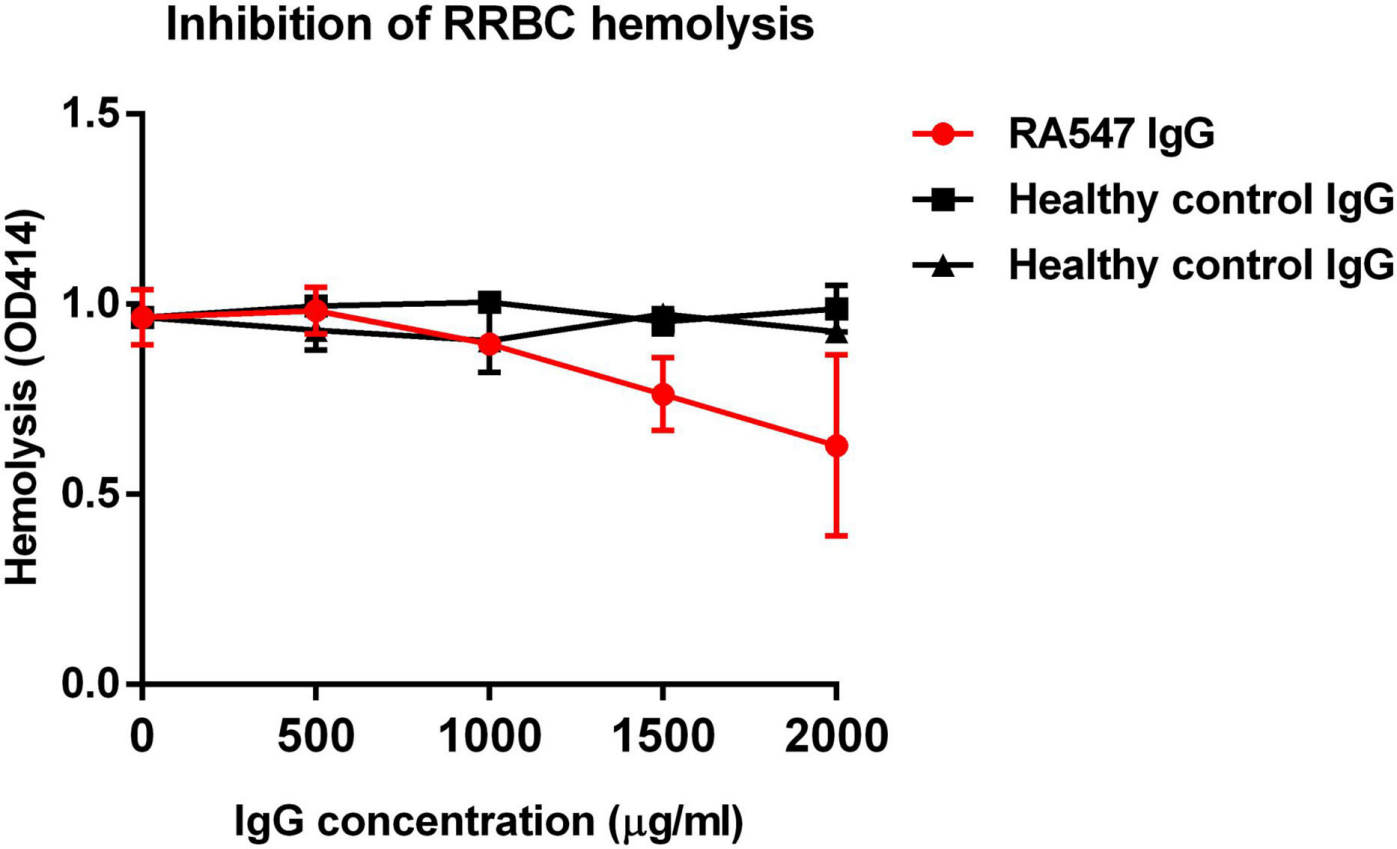
RA547 patient’s IgG dose-dependently inhibited RRBC hemolysis. Complement active normal human serum was mixed with RRBCs and IgG from patient or healthy controls, then incubated for 30 min at 37°C. Samples were centrifuged, and the OD of the supernatant was measured at 414 nm. Data are mean ± SD from two independent experiments.

To measure the effect of the purified, patient-derived anti-FB autoantibodies on the activation of the alternative pathway, rabbit red blood cells (RRBCs), which are sensitive to lysis in normal human serum, were used. RRBCs were mixed with normal human serum in a buffer containing Mg^2+^-EGTA, which allows only the activation of the alternative pathway, and supplemented with serial dilutions of IgG isolated from the serum of the patient or healthy controls. After incubation, lysis was determined by measuring the OD of released hemoglobin in the supernatant. Compared to the control IgGs, patient IgG inhibited the hemolysis in a dose-dependent manner (**Figure 5**).

**Figure 6:**
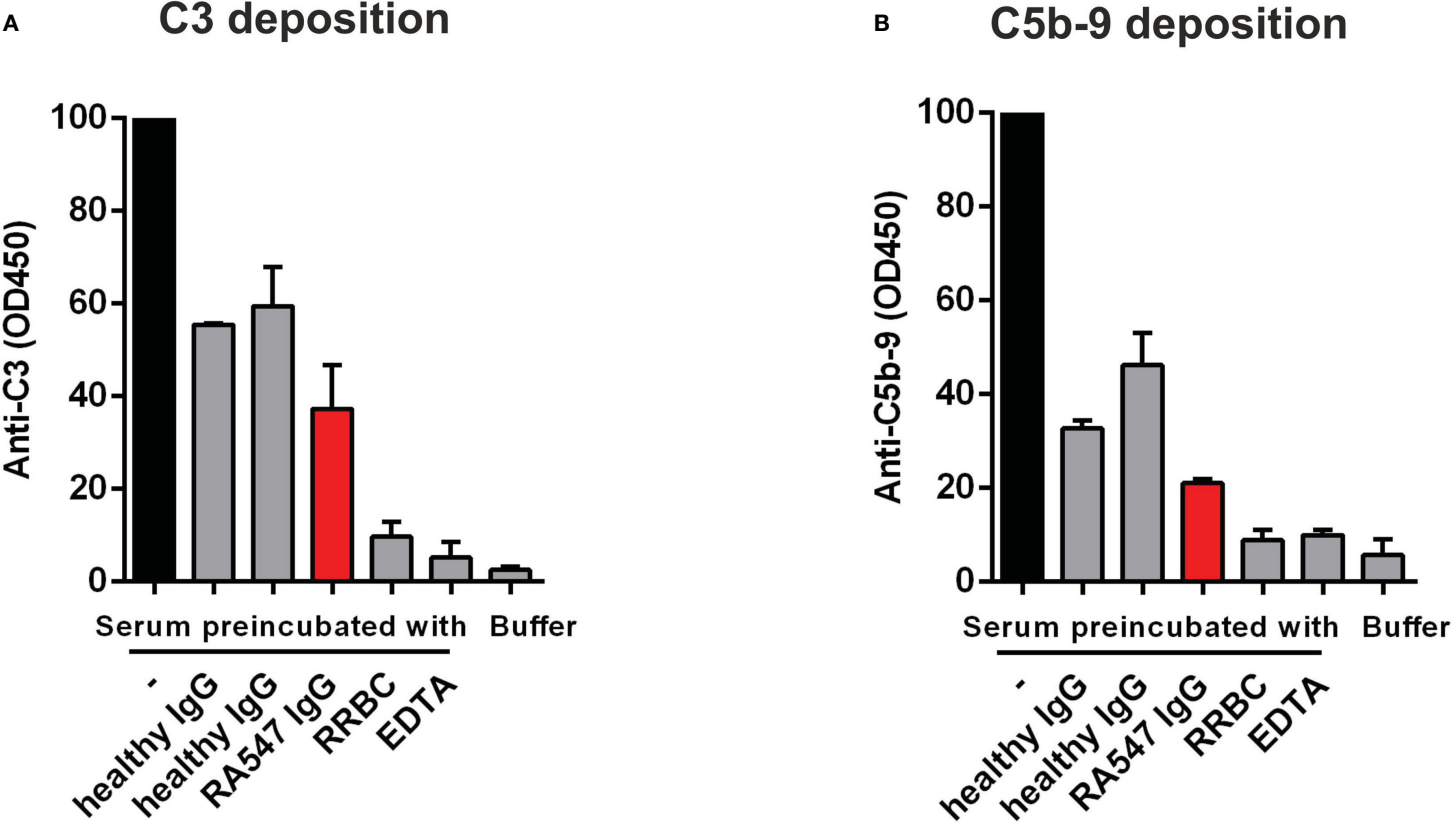
Anti-FB autoantibodies decrease complement activation-derived deposition of C3 and C5b-9 on LPS-coated surface. Normal human serum was preincubated with PBS, patient- or healthy control-derived IgG, and RRBC as a positive control, then added to LPS-coated wells. EDTA was used to inhibit complement activation on LPS. The deposition of **(A)** C3 and **(B)** C5b-9 was detected with anti-C3 and anti-C5b-9 antibodies, respectively. Data are mean ± SD from two independent experiments. RRBC: Rabbit Red Blood Cell.

Next, we investigated how the autoantibodies influence complement activation in serum in the fluid phase as described by Zhao et al. (38). To this end, normal human serum was mixed and incubated with IgG from RA547 or healthy controls and with RRBC as positive control. After incubation, the residual complement activity was measured on LPS-coated surface in the form of C3- and C5b-9-deposition (**Figure 6**). Serum alone retains its complement activity and when added to LPS, C3- and C5b-9-depostion can be detected. RRBC depletes complement during incubation, so no complement deposition can be measured on LPS, similar to the EDTA-treated serum sample. Incubation of the serum with IgG generally led to activity loss to some extent, probably due to activation *via* immune complexes present in the IgG preparations. However, compared to the IgG of healthy individuals, the autoanti-FB-containing patient IgG further decreased both C3- and C5b-9-deposition, presumably *via* inhibitory effects of the anti-FB autoantibodies (**Figure 6**).

### FB-autoantibodies impair the activity of the solid phase C3 convertase of the alternative pathway

**Figure 7:**
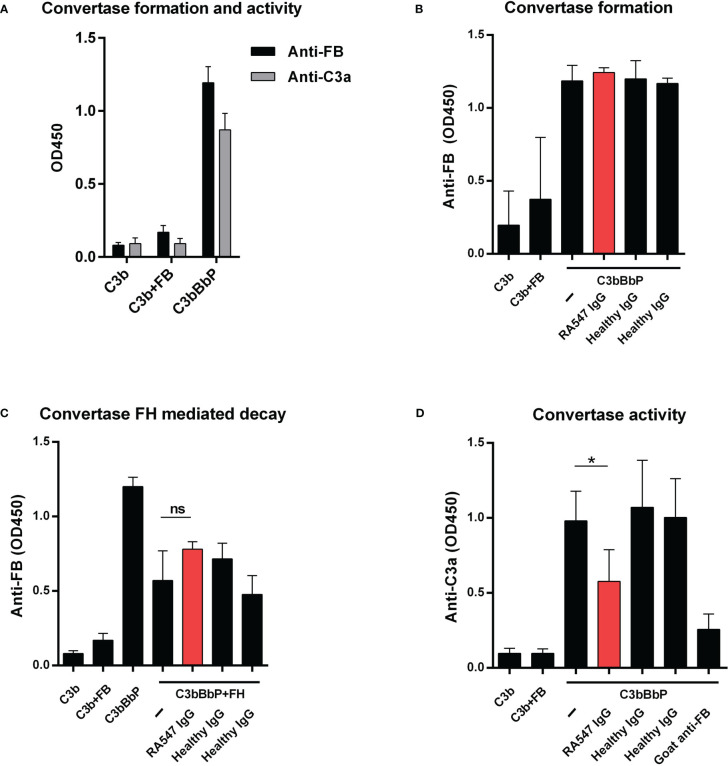
Effect of the patient’s IgG on alternative pathway C3 convertase. **(A)** Generation of solid phase C3 convertase in microplate wells. We added FB, FD and properdin together to the immobilized C3b and, after incubation, detected the convertase formation by polyclonal anti-FB. To confirm the activity of the convertase, C3 was added in the fluid phase, and we measured the released C3a from the supernatant with commercially available C3a EIA (Quidel). **(B)** To test the autoantibodies’ effect on convertase formation, we added the patient’s or healthy controls’ IgG together with FB, FD and properdin to immobilized C3b. The assembly of the convertase was detected with polyclonal goat anti-FB antibody. **(C)** Autoantibodies had no effect on the FH mediated decay of the C3 convertase. The convertase was incubated with IgG and FH and the remaining convertase was detected with polyclonal anti-FB antibody. **(D)** To investigate the effect of the patient’s autoantibodies on the activity of the convertase, the convertase was incubated with purified IgG and, after washing, C3 was added. Supernatants were collected and the C3 cleavage product C3a was measured using C3a EIA. Data are mean ± SD of three **(A, C, D)** or two **(B)** independent experiments. *p<0.05, One-Way ANOVA.

To decipher the mechanism behind the inhibitory effects measured using whole serum, next we analyzed the effect of the autoantibodies on the C3 convertase. C3bBbP was generated on microplates using purified proteins as described by Hourcade et al. (37) and Strobel et al. (39) with modifications. Convertase formation was detected with polyclonal goat anti-FB, while convertase activity was determined by adding C3 and measuring the generated C3a (**Figure 7A**).

First, we investigated the effects of autoantibodies on convertase formation. IgG from the patient and healthy controls were added to immobilized C3b together with the components of the convertase and, after incubation, convertase formation was measured. The anti-FB autoantibodies did not influence the assembly of the C3bBbP convertase (**Figure 7B**).

Next, we measured the effects of the autoantibodies on the FH-mediated decay of the convertase. The generated convertase was preincubated with IgG from the patient or healthy controls, then FH was added without removing the antibodies. After incubation, the remaining convertase was detected with goat anti-FB. The anti-FB autoantibodies had no effect in this assay; they did not protect the convertase from FH-mediated decay (**Figure 7C**).

Lastly, the effect of autoantibodies on convertase activity was measured. The generated convertase was preincubated with IgG from the patient and healthy controls, then C3 was added and the released C3a was measured from the supernatant. When added to the convertase, IgG of healthy controls had no effect, whereas, anti-FB autoantibodies significantly reduced the amount of generated C3a, similarly to that observed with the polyclonal anti-FB, used as positive control (**Figure 7D**).

### FHR1 and FHR3 deficiency

Deficiency of FHR1 and/or FHR3 is associated with various conditions, it can be both a protective and a predisposing factor depending on the disease (22, 40). We investigated the frequency of FHR1 and FHR3 deficiency in this RA cohort. Genomic DNA was not available; therefore, serum samples were screened for the presence of the FHR1 and FHR3 proteins by Western blot. To our knowledge, none of the patients or controls received plasmapheresis or fresh frozen plasma, thus the detected proteins have no *ex vivo* origin. We found individuals lacking only FHR1, only FHR3 or both proteins, both among the RA patients and the healthy controls, and the differences between the two groups were not significant (**Table 4**; **Supplementary Figure 2**).

**Table 4:** Overview of FHR1- and FHR3-deficiency in the RA patients and healthy controls.

Sample	N	FHR3 deficient	FHR1 deficient	FHR3/FHR1 deficient
RA	94	4 (4.25%)	1 (1.06%)	5 (5.31%)
Healthy control	117	2 (1.71%)	3 (2.56%)	4 (3.42%)

## Discussion

In this study we analyzed the presence of autoantibodies against complement components in a Hungarian RA cohort. We found anti-C1q and anti-MBL autoantibodies (**Figure 1**.), in accordance with previous reports (32, 33, 35). No antibodies against FH, FI, C3b and the alternative pathway C3 convertase (C3bBbP) were detected in these samples. In the case of FH, our results contradict the findings of Foltyn Zadura et al., who found FH-autoantibodies in 16.5% and 9.2% of the analyzed samples (34). While high IgG signals on FH-coated wells in 6.9% of samples were detected in our assays, these serum samples gave high signal on inert control protein as well, thus we considered them false positives. In addition to anti-C1q and anti-MBL autoantibodies, we found anti-FB autoantibodies in 5 patients, which have not been described in RA so far. The anti-FB IgG titer varied between patients; however, the presence of both light chains and various heavy chains (**Table 3**) was a common feature, suggesting an oligoclonal origin of the antibodies in all cases. Since the RA group and the healthy control group significantly differed regarding age and gender in our study, we cannot state that the identified autoantibodies are RA-specific. However, to our knowledge, no anti-FB autoantibodies have been described in healthy people at any age.

The analyzed anti-FB autoantibodies bind FB both in its native form and as part of the C3 convertase (**Figure 3C**). In contrast to C3Nefs that bind to and stabilize the C3 convertase, increase its activity and enhance terminal pathway activity (41), the analyzed anti-FB autoantibody did not influence solid-phase C3 convertase stability but impaired its activity (**Figure 7**). This is also different from the characteristics of the anti-FB autoantibody described in dense deposit disease (DDD) by Strobel et al. that stabilized the C3 convertase, increased its activity but inhibited the C5 convertase (39). FB autoantibodies described by Chen et al. in DDD and Marinozzi et al. in C3G also appeared pathogenic by enhancing C3 turnover (42, 43). Here, the anti-FB autoantibody binding site was mapped to the enzymatically active Bb part on FB (**Figure 4D**). Presumably, the autoantibody binding to Bb in the C3 convertase leads to impairment of C3 convertase activity measured in ELISA and in hemolysis assay (**Figures 7D**, **5**).

Since our Western blot analysis demonstrated the presence of FB within the isolated patient IgG fraction (**Figure 3D**), we conclude that the autoanti-FB antibodies formed immune complexes in the patient *in vivo*. We observed impaired complement activation in the form of reduced C3 and C5b-9 deposition on LPS-coated surface after preincubation of the serum with purified patient IgG, containing the FB–autoanti-FB immune complexes (**Figure 6**). This could be explained by activation of the classical pathway by the immune complexes and/or by the inhibitory effect of the anti-FB autoantibodies on C3 convertases formed on the LPS surface.

The production of such autoantibodies may be triggered by the prolonged presence of the complement protein activation products and complexes due to overactivation of the system. Our data demonstrate that not all the complement specific autoantibodies are pathogenic and contribute to complement activation in RA. Autoantibodies may be beneficial for patients, as it was shown for autoantibodies against FH in non-small cell lung cancer where anti-FH blocked FH activity at tumor cells (44) or in lupus patients (45). The autoanti-FB in RA reported here also demonstrates that some autoantibodies could hold back the overwhelming complement activation which may be beneficial for the patients. This suggests that the generation of such non-activating autoantibodies is part of a protective response and contributes to the prevention of the tissue damage caused by excessive complement activation.

The data also highlight that the presence/level of autoanti-FB in RA can be transient (**Figure 4A**) probably depending on the actual state of the disease (remission, or active phase), and the received treatment. In patient RA547 the autoanti-FB levels were mostly constant between May 2014 and November 2016, during which the patient had anti-TNFα therapy (certolizumab pegol). Later the therapy was changed (from 2020) to adalimumab which is another kind of anti-TNFα treatment. In the most recent serum sample of the patient (February 2022) autoanti-FB was not detectable, presumably due to the change in therapy or disease activity.

Autoantibodies against the components of the convertase can disturb the balance of complement activation, contributing to autoimmune diseases (39, 46). In many cases, autoantibodies have diagnostic and prognostic value, therefore their detection is coming to fore (47). Though in RA autoantibodies against the early components of the complement system (32, 33, 35) as well as autoantibodies against the complement regulator FH (34) were described years ago, their role in disease mechanism has not been investigated. Our results further support that the pathophysiology of RA implies the complement system and its dysfunction. These data also draw attention to the importance of characterization and functional analysis of autoantibodies, because simply detecting autoantibodies can be misleading and reporting their presence may misinform physicians. FB autoantibodies may be pathogenic in certain conditions, such as likely in DDD, but may be non-pathogenic or even protective in other diseases.

## Data availability statement

The original contributions presented in the study are included in the article/**Supplementary Material**. Further inquiries can be directed to the corresponding author.

## Ethics statement

The studies involving human participants were reviewed and approved by National Ethical Committee (33986-1/2018 EKU). The patients/participants provided their written informed consent to participate in this study.

## Author contributions

ATM and BU performed experiments, analyzed the data, prepared the figures. AF, BR, GN took care of the patients and provided serum samples. GS, MJ and BU initiated the study, supervised the research. ATM, BU and MJ wrote the manuscript with help of the other authors. All authors read and approved the manuscript. All authors contributed to the article and approved the submitted version.
