# Supplementary material for: Autoantibodies against complement factor B in rheumatoid arthritis
Source: Front Immunol. 2023 Feb 20;14:1113015. doi: 10.3389/fimmu.2023.1113015 (PMC9986603; doi:10.3389/fimmu.2023.1113015)
Supplement: Supplementary file 1 [file DataSheet_1.pdf]

Supplementary Material

Autoantibodies against complement Factor B in rheumatoid arthritis

Alexandra T. Matola[1,2], Angéla Fülöp[3], Bernadette Rojkovich[3], György Nagy[4,5,6], Gabriella Sármay[1], Mihály Józsi[1,2,†,*], Barbara Uzonyi[1,2, †]

[1]Department of Immunology, ELTE Eötvös Loránd University, Budapest, Hungary

[2]MTA-ELTE Complement Research Group, Eötvös Loránd Research Network (ELKH) at the Department of Immunology, ELTE Eötvös Loránd University, Budapest, Hungary

[3]Buda Hospital of the Hospitaller Order of Saint John of God, Budapest, Hungary

[4] Department of Rheumatology and Clinical Immunology, Department of Internal Medicine and Oncology, Semmelweis University, Budapest, Hungary

[5] Heart and Vascular Center, Semmelweis University, Budapest, Hungary

[6] Department of Genetics, Cell- and Immunobiology, Semmelweis University, Budapest, Hungary

[†] These authors contributed equally to this work and share last authorship

*** Correspondence:**
Mihály Józsi (mihaly.jozsi@ttk.elte.hu)

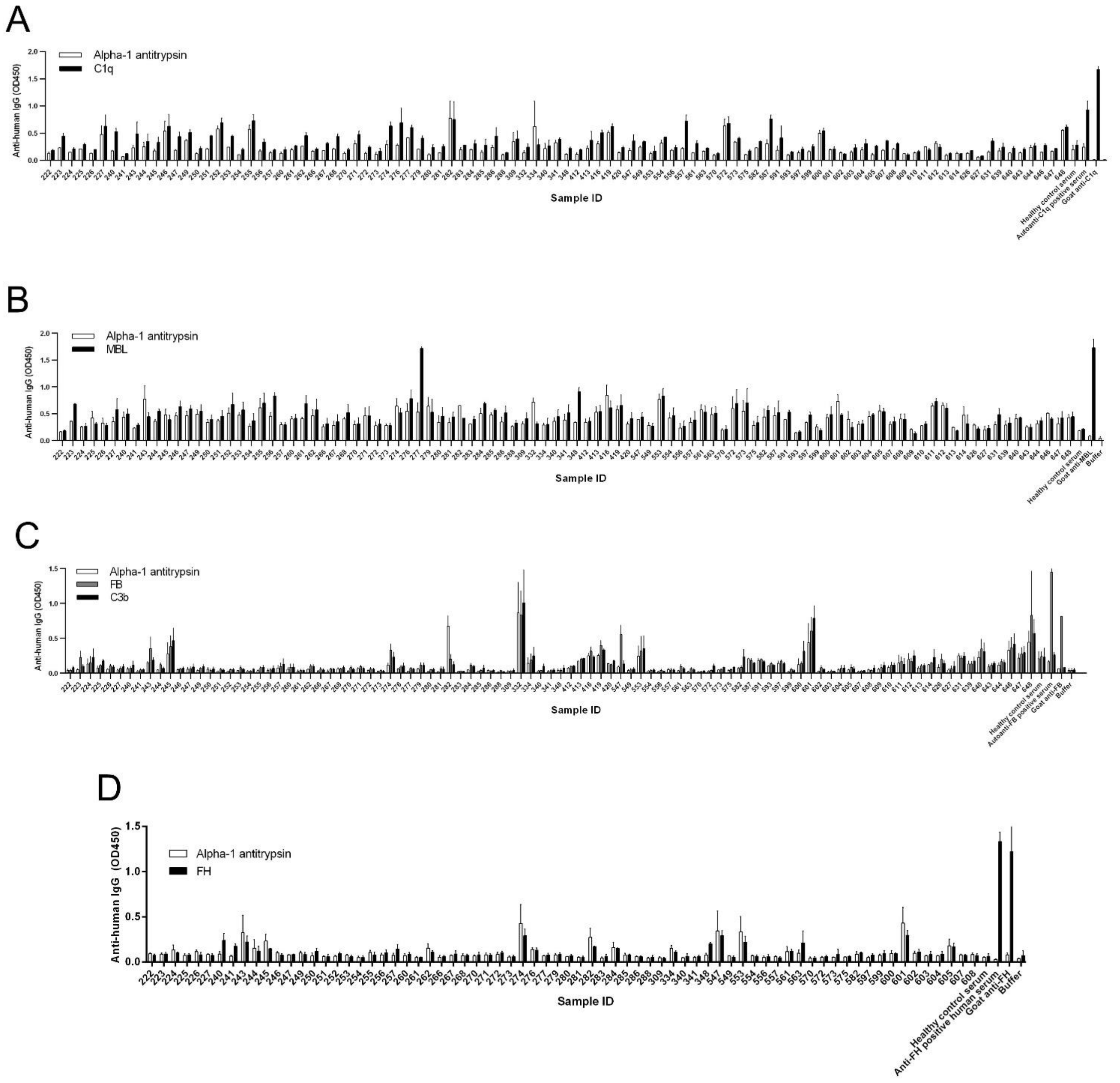

Supplementary Figure 1. Autoantibodies against C1q, MBL, FB and FH were analysed in RA patients. (**A**) C1q, (**B**) MBL, (**C**) FB and C3b and (**D**) FH, and as negative controls alpha-1 antitrypsin were immobilized in microtiter plate wells. After blocking, the wells were incubated with serum samples diluted 1:50. Bound IgG was detected by anti-human IgG.

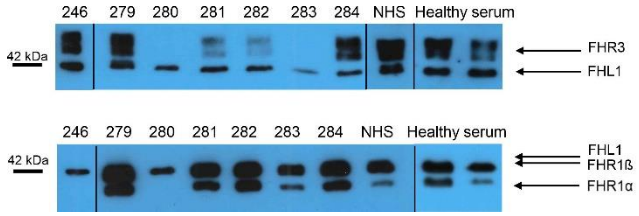

Supplementary Figure 2. Detection of FHR1 and FHR3 deficiency by Western blot. Serum samples were separated on 10% SDS-PAGE under non-reducing conditions. After transferring the proteins to nitrocellulose membrane, the presence of FHR3 (top) and FHR1 (bottom) was detected with polyclonal anti-FHR3 and polyclonal anti-FH, respectively. Both antibodies recognize FHL1 as well. Pooled normal human serum (NHS) was used as a positive control containing both FHR1 and FHR3. Representative blots depicting FHR1- (#246), FHR3- (#283) and combined FHR1-FHR3-deficiency (#280), as well as nondeficient cases are shown.
